# On the Use of Strychnia as a Remedy for Intermittent Fever; Being the Substance of a Clinical Lecture Delivered at the Chicago Hospital, May 1st, 1847

**Published:** 1847-06

**Authors:** Daniel Brainard

**Affiliations:** Professor of Surgery in Rush Medical College


ARTICLE IV.

### *On the Use of Strychnine as a Remedy for Intermittent Fever, being the Substance of a Clinical Lecture delivered at the Chicago Hospital, May 1st,* 1847. By DANIEL BRAINARD, M. D., Professor of Surgery in Rush Medical College.

Those who have been in attendance at the College Dispensary during the past winter, as well as those who are now following the visits of this establishment, must often have their patience wearied by the number of cases of old intermittents which present themselves, and which, in spite of the best directed treatment we have been enabled to give them, had relapsed, and returned from time to time for asstance. For ourselves, far from being wearied with this great number of cases, thought by many uninteresting, we have subjected them to the most careful examination in order to determine their causes and pathology, and have conducted a series of careful trials in order to determine the value of different remedial agents in their treatment.

In reference to the cause of intermittents, notwithstanding their extreme prevalence and the great number of observers who have directed their attention to it, it is as much involved in doubt as ever. The agency of "malaria" is doubted, and, as it appears to us, with great reason; for a number of these fevers are produced, and all of them may readily be reproduced, in situations where the agency of such a substance cannot be suspected. Nor have the other theories advanced to account for its production appeared more reasonable. The only circumstance which has seemed to us to be invariably present at its production, in every individual *in the first instance*, is an elevated temperature, whose degree we cannot, at this moment, specify with certainty, but which is probably about 80° of Fahrenheit's thermometer, and which requires to be continued for a certain length of time. But whether this acts directly upon the system or through the medium of some exhalation or miasm, we are ignorant.

In regard to its pathology, a careful observation of a great number of cases has led us to the following conclusions:

1st. The first link in the chain of morbid actions constituting periodical fever consists in a derangement of the digestive functions, indicated by furred tongue, loss of appetite, flatulence, deranged action of the bowels, &c.

2d. The languor, lassitude, sense of weariness and inactivity of all the faculties, considered by Smith and others as the first perceptible deviation from healthy action, is but secondary to the imperfect assimilation.

3d. Soon after the occurrence of the second class of symptoms there is a perceptible change in the blood, the fibrine being, as in all other fevers, diminished in quantity.

4th. These, with general derangement of all the secretions constitute as far as we have been able to observe the uniform symptoms of fever before the occurrence of a paroxysm.

5th. Persons thus affected may induce a paroxysm of intermittent fever by exercise, exposure to cold, mental action, eating indigestible substances, &c. Whatever acts with even a moderate degree of force in deranging the functions of the system is capable of inducing the paroxysm. In considering the treatment of these persons it is important to bear in mind that the state of debility and deranged actions of all the organs precede, in all cases, to some degree, the occurrence of chills and their sequelæ, and that when these latter are arrested, the same state which preceded them still persists, often in an increasing degree. So that it becomes necessary in speaking of the treatment to separate the means calculated to interrupt the paroxysms, from those capable of restoring the energies of the body, and preventing their recurrence.

This distinction has, it is true, been made by medical writers generally, but not to the same extent as among the people, where the "breaking the fits" and curing the disease are generally distinguished from each other.

In a considerable number of cases the same causes which induce the paroxysms in the first instance, will serve to arrest them. Thus sudden fright, a shower bath, terror, electrical shocks, the inhalation of ethereal vapor, intense mental application, &c., are capable of arresting the periodical accessions temporarily. There are a large number of medicines capable of effecting the same object more certainly and for various periods of time. Emetics, cathartics, sudorifics, narcotics, stimulants, and tonics have been used. Specifics or febrifuge medicines, whose mode of action is unknown, are more frequently relied on. Under this head quinine, arsenic, salicine, piperine, prussiate of iron, and strychnine, are the most prominent. The sulphate of quinine is an article so safe and so uniformly efficacious, that if the arrest of the paroxysm were all that were required we should, in the greater number of cases, have occasion to seek for no other remedy. But unfortunately it generally exerts but little other influence over the system than of arresting them, and they return with great regularity usually in from seven to fourteen days. In proportion to the number of returns the quinine loses its influence, so that after taking it, as most of the patients who present themselves here have done, from ten to twenty-times, its effect is very slight; a large dose often failing entirely to check the disease. On this account, and from the inutility of wasting large quantities of the medicine, we have been obliged to search for other means as substitutes for it, in these cases. The arsenial solution has been tried in a considerable number of them, and it has been found to succeed in most instances where the quinine failed and is in general also more permanent. But sooner or later the disease returns after its use, and the state of debility upon which these old cases of ague attend, is not in any degree removed by its use.

The ordinary form of using the quinine has been, with us, to give it in doses of from five to ten grains repeated once or twice daily till ℈j or ℈jss has been administered. In using the arsenial solution one grain was used in sixteen doses during forty-eight hours, at equal intervals. Finding the cases to return regularly in spite of their use, we have sought other means. The trials of salicine made at the New Orleans Charity Hospital and elsewhere, show that nothing is to be expected of it in these cases, although it is worthy of a trial in other diseases. Piperine is still less useful, and does not, according to our observations, possess any advantages over pulverized capsicum, which frequently is a useful adjuvant to the quinine. The prussiate of iron has also been tried either alone or in combination with quinine, but it is without efficiency in severe cases, and possesses no advantages over the quinine where it succeeds. It will be readily perceived, from this brief review of the principal means we are in the habit of using against agues, that there is an urgent necessity for researches directed with a view of finding a substitute for the quinine or some means to be used in aid of its action; and this necessity will appear the greater when we take into consideration the fact that nearly all the laboring population of this State have been affected by the ague and that, after a few returns, the larger number are unable to purchase the quinine in consequence of its high price. It is probable that the inhabitants of Illinois have, during the past year expended not less than from $100,000 to $200,000 for different ague medicines.

Profiting by the opportunity afforded in a public institution, we have determined to make trial of a medicine which has been often recommended but scarcely ever used in this country for the cure of intermittents: namely, the strychnine. Our attention, was directed to this substance from the manifest analogy between its effect and those of quinine, from the benefit which has been found to result from its use in chronic diarrhœa and debility of the digestive organs, and from the powerful tonic effect it is known to exercise over the whole system, but especially the nervous and muscular systems. The result has been most satisfactory. If it has not exhibited, in every case, the promptness of quinine in arresting the paroxysm, it has yet done so in four-fifths of them in which it has been used, while in the permanence of its action it has more than counterbalanced any defect in promptness. Indeed, it is highly probable that this apparent defect of action in some cases may be owing to the caution with which it was given, since, sometimes it was found to succeed in cases where quinine in full doses had failed.

The mode of administration is as follows: one-eighth of a grain is given thrice daily, after meals, in form of powder or pill until one grain is taken, mixed, in either case, with flour or starch. If pills are used, they should be recently made and not allowed to become dry and hard. There is an advantage in giving it after meals, as it then becomes mixed with the contents of the stomach, and is less likely to produce unpleasant effects. Smaller doses were at first used without effect.

It is given in combination with cathartics when the bowels are costive, with diaphoretics when there is much reaction, and in general combined with the same medicines as the quinine.

*Effects.*—In much the larger number of cases the patient is not conscious of any sensible effect, except the arresting of the fever and a sensation of well-being that is usually experienced for two or three days after its use has been discontinued. In a smaller number of cases, the patients stated that it produced intoxication; and in a still smaller number, vertigo, with pain in the head and nausea were experienced. In order to illustrate its effects, we will relate a few cases where it operated favorably, choosing those which are best calculated to exhibit its powers under different circumstances.

Case I.—I. F., Æt. 40 years, had, in August, 1846, a severe remittent fever, from which he entirely recovered, except that there was some weakness remaing. When he attempted to work he was attacked with intermittent fever, and this continued to recur during the winter, being usually checked by quinine, of which one scruple would arrest it for from two to three weeks, but he was too unwell during the whole winter to do a day's work in one day. February 25th, he commenced taking strychnine one-eighth grain three times daily till one grain had been taken, and then the same quantity every third day for sixteen days. Since which time to the present, May 1, 1847, he has had no return of paroxysm, but has pursued a laborious occupation, and says he never felt better.

Case II.—I. S., a servant girl, had, in the month of July, 1846, an intermittent fever which has continued to recur for seven months, every two or three weeks being arrested by quinine and various patent preparations. March 1st, she commenced taking the strychnine, one-eighth grain thrice daily till one grain was taken. Since which time she has had no return of disease, but says she feels well in every respect.

It would be easy to multiply these cases, but they present such similarities that it would be but a repetition of the same thing. I will therefore give you the result of its administration in the college dispensary up to the first of April, 1847, its use having been commenced about the 20th of February. I reject the cases treated in this hospital and in private practice since that date, as the time has as yet been too short to judge of its permanent influence in these cases. Up to the first of April, 1847, the strychnine was prescribed in the way described in case No. 2, in eighty-three cases.

| | |
|---|---|
| Of these it had no influence over - - | 14 cases. |
| It arrested the paroxysms for one week in - | 3 cases. |
| For two weeks in - - - - - | 6 cases. |
| It arrested it permanently, or till May 1st, in - | 60 cases. |
| Making the total of - - - | 83 cases. |

The cases in which it is put down as permanently arrested are those in which there has as yet been no return. Of the above, two cases had taken the quinine without the smallest effect, but were relieved by the strychnine. Nearly all, and probably every one, had taken the quinine from time to time with only temporary relief. All were old cases in which there had been from two to twenty returns of the disease in the course of twelve months. The cases in which its effects were most favorable were those where there was no local inflammation or disease. Those where it was least useful were cases complicated with dropsy, chronic bronchitis, or phthisis; for this latter is, after all that has been said to the contrary, a very frequent effect of ague. When the chills occur in the evening it indicates a tendency to hectic and the medicine is then unsuccessful.

*Dangerous and Inconvenient Effects of Strychnine.*—In but three of the eighty-three cases above stated, were the symptoms of nausea and vertigo present. It is, however, obvious that so active a medicine cannot be entrusted to ignorant people without dangerous effects sometimes following its use. Thus, an individual who forgot to take his pill after breakfast, made up for it by taking two pills after dinner, and had as the effects of this dose (one-fourth grain) severe spasms in the legs. Another, thinking if one would do good more would do better, took six pills at once, and his wife, who seemed to think it an excellent joke, said it cramped him "all in a heap." A third was asked by a physician what she was taking, when she showed him the powders. Taking it for quinine, probably, and forgetting the rules of medical ethics as well as the dictates of prudence, he called for a cup and gave her six at a dose. She was affected with severe cramps, which he mistook for hysteria and administered tinct. assafœtida. She recovered without other ill effects. These cases, while they show the necessity of caution, show also that it is less dangerous than is supposed by many. In order, however, to obviate the danger we have lately adopted the practice of combining gr. $\frac{1}{8}$ of it with gr. xx of starch, and gr. ij of pulv. ipecacuanha. From the bulk and nauseous taste there is little danger of a person taking more than one dose, and if several were swallowed at a time the effect would be to produce vomiting. In fact this dilution of the medicine reduces it to about the strength of quinine, for which it is usually mistaken by patients, and hence takes away the principal danger of its use.

From the facts and cases here stated we are justified in concluding that, in a very large number of cases of ancient agues attended with debility, and unaccompanied by local inflammation, the strychnia is very nearly equal to the quinia in arresting the paroxysms, and much superior to it in removing that state of debility and derangement of the secretions, which we have already stated to constitute the first and persisting pathological state of the disease. Considered merely in an economical point of view, its use would be a great gain, for one grain is found in the cases in which it succeeds to equal in efficiency from a scruple to a drachm of quinine. As a useful auxiliary and, in some cases, as a substitute for quinine, it seems to promise favorably, and we lay these observations before the profession in the hope that by further and various trials, the form and manner of its administration and the cases and circumstances in which it is useful will be accurately determined.

